# Improved Healing of Diabetic Foot Ulcer upon Oxygenation Therapeutics through Oxygen-Loading Nanoperfluorocarbon Triggered by Radial Extracorporeal Shock Wave

**DOI:** 10.1155/2019/5738368

**Published:** 2019-08-14

**Authors:** Shunhao Wang, Chunyang Yin, Xiaoguang Han, Anyi Guo, Xiaodong Chen, Sijin Liu, Yajun Liu

**Affiliations:** ^1^State Key Laboratory of Environmental Chemistry and Ecotoxicology, Research Center for Eco-Environmental Sciences, Chinese Academy of Sciences, Beijing 100085, China; ^2^University of Chinese Academy of Sciences, Beijing 100049, China; ^3^Beijing Jishuitan Hospital, The 4th Clinical Hospital of Peking University Health Science Center, Beijing 100035, China; ^4^School of Aerospace Engineering, Beijing Institute of Technology, Beijing 100081, China

## Abstract

Diabetic foot ulcers (DFUs), the most serious complication of diabetes mellitus, can induce high morbidity, the need to amputate lower extremities, and even death. Although many adjunctive strategies have been applied for the treatment of DFUs, the low treatment efficiency, potential side effects, and high cost are still huge challenges. Recently, nanomaterial-based drug delivery systems (NDDSs) have achieved targeted drug delivery and controlled drug release, offering great promises in various therapeutics for diverse disorders. Additionally, the radial extracorporeal shock wave (rESW) has been shown to function as a robust trigger source for the NDDS to release its contents, as the rESW harbors a potent capability in generating pressure waves and in creating the cavitation effect. Here, we explored the performance of oxygen-loaded nanoperfluorocarbon (Nano-PFC) combined with the rESW as a treatment for DFUs. Prior to *in vivo* assessment, we first demonstrated the high oxygen affinity *in vitro* and great biocompatibility of Nano-PFC. Moreover, the rESW-responsive oxygen release behavior from oxygen-saturated Nano-PFC was also successfully verified *in vitro* and *in vivo*. Importantly, the wound healing of DFUs was significantly accelerated due to improved blood microcirculation, which was a result of rESW therapy (rESWT), and the targeted release of oxygen into the wound from oxygen-loaded Nano-PFC, which was triggered by the rESW. Collectively, the oxygen-saturated Nano-PFC and rESW provide a completely new approach to treat DFUs, and this study highlights the advantages of combining nanotechnology with rESW in therapeutics.

## 1. Introduction

Diabetic foot ulcers (DFUs), the most common complication of diabetes mellitus, are caused by peripheral neuropathy, small vessel occlusion, and secondary infection or trauma, and they may lead to lower extremity amputation [1–4]. Many adjunctive strategies have been developed for the treatment of DFUs in the clinical practice, including negative pressure wound therapy, ultrasound, recombinant human platelet-derived growth factor-BB, and acellular matrix products [5–7]. However, low efficiency, potential side effects, and high cost have limited their wide application. Hypoxia is a key inhibiting factor for wound healing of DFUs, which can block fibroblast proliferation, collagen production, and capillary angiogenesis and enhance the risk of infection [8–10]. Hyperbaric oxygen therapy (HBOT) is the most commonly utilized adjunctive therapy for improving wound tissue hypoxia in DFU treatment [11–13], but this method has not achieved universal success in many studies and is costly [14, 15]. And the production of HBO-related oxidative stress is a concern [16]. Thus, to improve treatment efficiency and reduce costs, a need exists for a new and effective DFU treatment method.

A nanomaterial-based drug delivery system (NDDS) could improve DFU treatment by means of targeted drug delivery and controlled drug release [17–19]. Recently, growing attention in the NDDS has given rise to the study of micro/nanobubbles, especially oxygen-filled nanobubbles [8, 20–22]. Among them, nanoperfluorocarbon (Nano-PFC) has been extensively explored as an oxygen-loaded system to overcome hypoxia-associated resistance in cancer therapies owing to its high oxygen affinity and great biocompatibility [22–24]. Additionally, oxypherol (Fluosol-43), a type of PFC, has obtained US Food and Drug Administration (FDA) approval for improving myocardial oxygenation and preventing abnormalities in ventricular function [25]. Thus, Nano-PFC can be used as an NDDS to deliver molecules, such as drugs and oxygen, to target tissues and release the contents in response to physical stimuli [20, 23, 26].

The radial extracorporeal shock wave therapy (rESWT) has been widely applied in musculoskeletal disorders, myocardial infarction, wound healing, and erectile dysfunction due to the noninvasive mode of treatment, cost effectiveness, lower energy level, and negligible side effects [27–29]. More importantly, rESW is a type of pneumatically generated pressure wave [30, 31] that can induce the cavitation effect, which refers to the rapid formation, expansion, and collapse of small vapor bubbles in liquids due to sharp pressure changes [32–34]. Thus, the rESW is a candidate trigger source to enhance inclusion release inside an NDDS. Furthermore, ESW therapy has been reported to distinctly improve and promote the healing of DFUs by mainly enhancing blood microcirculation and tissue regeneration and reducing oxidative stress [35, 36]. However, the corresponding studies about rESWT are few.

Herein, we developed a completely new strategy for the treatment of DFUs by combining the rESW with oxygen-loaded Nano-PFC, which could effectively improve blood flow and provide a targeted supply of oxygen. In our study, the rESW-responsive oxygen release feature of Nano-PFC was first proved *in vitro* and *in vivo*. Moreover, rESWT was demonstrated to improve blood microcirculation and accelerate the wound healing of DFUs. Based on our results, the rESW-responsive oxygen-loaded Nano-PFC can provide a new sight and great potential for DFU treatment.

## 2. Materials and Methods

### 2.1. Preparation and Characterization of Nano-PFC

PFC (300 *μ*L perfluoro-15-crown-5-ether, Fluorochem, UK) was added to 1% human serum albumin (HSA) (Sigma-Aldrich, China) in 0.01 M phosphate-buffered saline (PBS) solution (4 mL). Then, the mixture was emulsified by an ultrasonic homogenizer (Scientz-1200E, China) for 200 s after slight oscillation. The obtained emulsion was centrifuged (8000 r/min) for 3 min, and then, the precipitate was resuspended in PBS for further use. The size and morphology of Nano-PFC were characterized by transmission electron microscopy (TEM) (SU-8020, Hitachi, Japan) after negative staining using 1.5% phosphotungstic acid and Malvern zetasizer (NANO ZS, UK).

### 2.2. Measurement of Oxygen Release from Nano-PFC Triggered by rESW *In Vitro*

Nano-PFC solution (4 mL, containing 300 *μ*L of PFC) was stored in an aseptic oxygen chamber (O_2_ flow rate = 5 L/min) for 5 min for Nano-PFC oxygenation. The oxygen-free water was obtained after N_2_ bubble. Then, oxygen-saturated Nano-PFC was added to 45 mL of oxygen-free water. The oxygen concentration was measured by a portable dissolved oxygen meter (Rex, JPBJ-608, China) before and after adding Nano-PFC with or without rESW (MASTERPULS® MP100, STORZ MEDICAL AG, Switzerland) treatment (1 bar, 2 Hz, 25 min).

### 2.3. Cellular Experiments

Murine breast cancer (4T1) and human umbilical vein endothelial (HUVE) cells were purchased from American Type Culture Collection (ATCC) and cultured with 1640 medium containing 10% fetal bovine serum (FBS, HyClone) at 37°C in a 5% CO_2_ atmosphere. The 4T1 and HUVE cells were seeded into separate 96-well plates (0.8 × 10^4^ cells/well). After 24 h, the different amounts of Nano-PFC (0.25, 0.5, 1, 2, and 4 *μ*L) were added to the 96-well plates, and the cells were incubated for 24 h (*n* = 4). The cell viability was determined by the WST-8 (4-[3-(2-methoxy-4-nitrophenyl)-2-(4-nitrophenyl)-2H-5-tetrazolio]-1,3-benzene disulfonate sodium salt) assay following a standard protocol (Solarbio, 1000T, China).

### 2.4. Animal Model

All of the animal experiments were approved by the Animal Ethics Committee of the Research Center for Eco-Environmental Sciences, Chinese Academy of Sciences. Sixteen female albino Wistar rats (180–220 g) were purchased from the Peking University Laboratory Animal Research Center and randomly divided into Ctrl, rESW, Nano-PFC@O_2_, and rESW+Nano-PFC@O_2_ groups (*n* = 8). The rats in all groups were intraperitoneally injected with streptozotocin (STZ) solution (Sigma-Aldrich, 60 mg/kg body weight) that was freshly dissolved in 0.1 mg/L citrate-sodium citrate buffer (pH 4.5) for approximately 2 months. With respect to the blood glucose level, measured by a glucometer, it was ≥300 mg/dL for two consecutive weeks, suggesting that the STZ-induced diabetic rat model was successfully established, in agreement with previous reports [10]. In order to faithfully reflect the realistic complex pathologies and mimic those conditions under diabetes mellitus, we did not actually add additional treatments. Therefore, during this period, we kept monitoring blood glucose levels without additional work. Nonetheless, the protocol of establishing a STZ-induced diabetic rat model has been widely applied in the wound healing studies of diabetic foot ulcers, as supported by the following references (Table S1) [10–17]. Then, a wound (5 × 5 mm) was created in the left forepaw of each rat after anesthetizing with sodium pentobarbital (45 mg/kg body weight) by intraperitoneal injection. The size of the wound was measured by a Vernier caliper. BALB/c female mice (7-8 weeks old) were purchased from the Vital River Laboratory Animal Technology Co. Ltd. (Beijing, China). The 4T1 tumor-bearing mouse model was established following the instructions in our previous reports [37].

### 2.5. *In Vivo* Photoacoustic (PA) Imaging

When the tumor volume of 4T1 tumor-bearing mice reached up to 100 mm^3^, the tumor oxygenation levels were detected using a PA imaging system (Vevo 2100, FUJIFILM VisualSonics Inc., Canada) utilizing the oxy-hemo mode (750 and 850 nm). The 4T1 tumor-bearing mice were injected with oxygen-saturated Nano-PFC through the tail vein (200 *μ*L, containing 30 *μ*L of PFC), and the mice breathed pure oxygen for 20 min before PA imaging.

### 2.6. Blood Flow by Laser Doppler Imaging

The blood flow of the wound was measured by laser Doppler imaging (Moor LDI V3.01, UK). STZ-induced diabetic rats were injected with oxygen-saturated Nano-PFC through the tail vein (500 *μ*L, containing 80 *μ*L of PFC), and the mice breathed pure oxygen for 20 min before imaging. The rats in the rESW+Nano-PFC@O_2_ group were treated with rESW for 20 min (1 bar, 2 Hz). The quantitative data were obtained from Moor FLPI measurement software (Version 2.1).

### 2.7. Immunohistochemistry Analysis

After various treatments for 4T1 tumor-bearing mice, the mice were sacrificed and the tumors were isolated from the mice on the 15th day and then fixed in 4% paraformaldehyde, embedded, and sectioned at 8 *μ*m. Next, the immunohistochemical analysis of HIF-1*α* for tumor tissues was performed as the protocol. The expression levels of HIF-1*α* were quantified by ImageJ software (https://imagej.nih.gov/ij/).

### 2.8. Nano-PFC plus rESW Treatment *In Vivo*

The STZ-induced DFU rats were anesthetized with sodium pentobarbital (45 mg/kg body weight) by intraperitoneal injection prior to treatment. Thereafter, the rats were injected with PBS (500 *μ*L) through the tail vein for the control group. The rESW group received rESW treatment (1 bar, 2 Hz, 20 min). The rats were injected with oxygen-saturated Nano-PFC through tail vein intravenous injection (500 *μ*L, containing 80 *μ*L of PFC) and subjected to pure oxygen breath for 20 min in the Nano-PFC@O_2_ group. The rESW+Nano-PFC@O_2_ group denoted the rats that inhaled pure oxygen for 20 min after Nano-PFC@O_2_ injection (500 *μ*L, containing 80 *μ*L of PFC). Afterwards, the rats were treated with rESW (1 bar, 2 Hz) for 20 min. All administration and treatments were conducted once every other day for a total of three times. The size of the wound was measured by a Vernier caliper.

### 2.9. Statistical Analysis

The statistical analysis of experimental data was determined by an independent *t*-test or one-way ANOVA test using the SPSS Statistics 17.0 software. All of the data are presented as the mean ± standard error. Statistical significance was determined with *P* < 0.05 and *P* < 0.001.

## 3. Results and Discussion

### 3.1. Synthesis and Characterization of Nano-PFC

**Figure 1:**
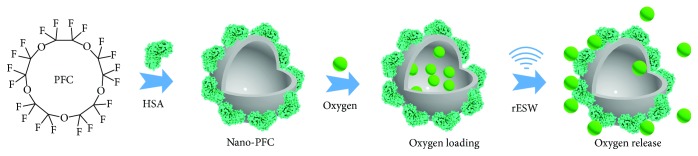
Schematic illustration of the synthesis procedure and rESW-responsive oxygen release from Nano-PFC.

**Figure 2:**
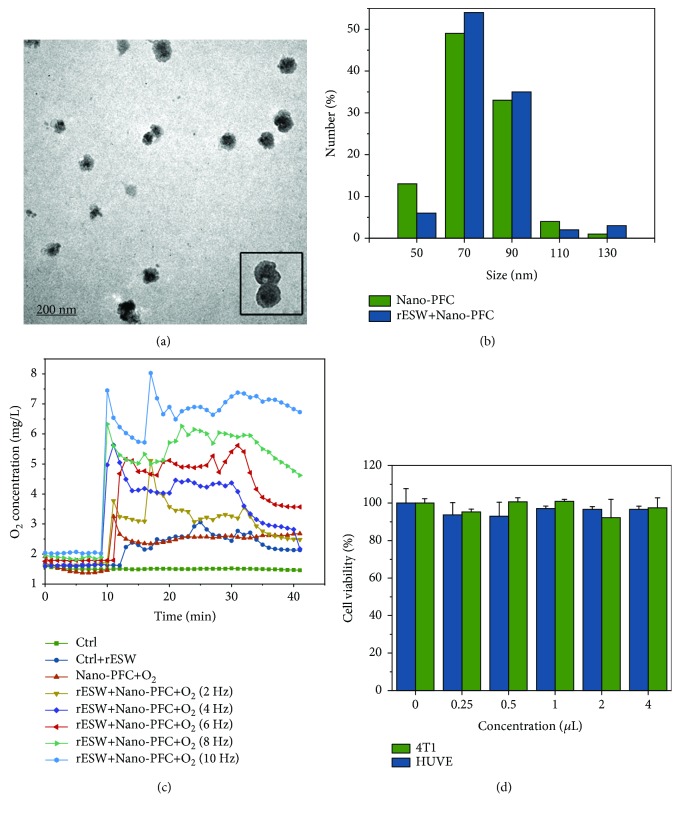
Synthesis and characterization of nanomaterials used in the current study. (a) TEM images of Nano-PFC, and an inset image shows the structure of Nano-PFC. (b) The size distribution of Nano-PFC before and after rESW treatment. (c) Time-/dose-dependent changes of dissolved oxygen concentrations in deoxygenated pure water with or without the addition of oxygen-loaded Nano-PFC@O_2_. The solutions were treated with rESW for 25 min. (d) Cell viability was measured with the WST-8 method of 4T1 and HUVE cells upon Nano-PFC at various concentrations (*n* = 4) for 24 h.

The nanodroplets of HSA-stabilized Nano-PFC were obtained using the modified microemulsion method under ultrasonication, as described in previous reports (Figure 1) [22, 23]. Figures 2(a) and 2(b) show that the synthesized HSA-stabilized Nano-PFC exhibits a highly uniform size distribution with an average diameter of about 70 nm, as measured by TEM and dynamic light scattering (DLS). Moreover, the size of Nano-PFC changed negligibly after rESW treatment, which proved that Nano-PFC had great stability (Figure 2(b)).

### 3.2. rESW-Responsive Oxygen Release of Nano-PFC *In Vitro* and *In Vivo*

PFC, as an artificial oxygen carrier, has been used in the clinical treatment of ischemic diseases such as hemorrhagic shock and allogeneic blood transfusions owing to its excellent dissolving capacity for many gases, including oxygen and carbon dioxide [25, 38]. Thus, the abilities of Nano-PFC for oxygen loading and rESW-responsive oxygen release (Figures 1 and 2(c)) were measured by an oxygen meter. The concentration of pure oxygen saturation within Nano-PFC was about 1.50 mg/mL at 25°C (1 atm), which was consistent with previously reported results [23]. As shown in Figure 2(c), the dissolved oxygen concentration in water speedily increased in a short time, and then, the oxygen was slowly released from the oxygen-loaded nanodroplets over time when the oxygen-loaded Nano-PFC was added to oxygen-free water. Importantly, burst-like oxygen release in a dose-dependent manner was observed under rESW treatment with different frequencies (2, 4, 6, 8, and 10 Hz). These results demonstrated the high oxygen solubility and rESW-responsive oxygen release abilities of Nano-PFC. Moreover, after one injection, Nano-PFC may reload oxygen within the lung capillaries during blood circulation. Thus, oxygen-filled Nano-PFC can deliver oxygen to the targeted area and then reversibly release oxygen triggered by the rESW. Compared to HBOT, this Nano-PFC-based treatment strategy can effectively reduce the cost of treatment and avoid serious side effects, such as barotrauma, central nervous system and pulmonary oxygen toxicity, and increased risk of claustrophobia [39]. In addition, the cell viability of 4T1 and HUVE cells was not significantly affected by the different doses of Nano-PFC, which exhibited that Nano-PFC has great biocompatibility for different types of cells (Figure 2(d)).

**Figure 3:**
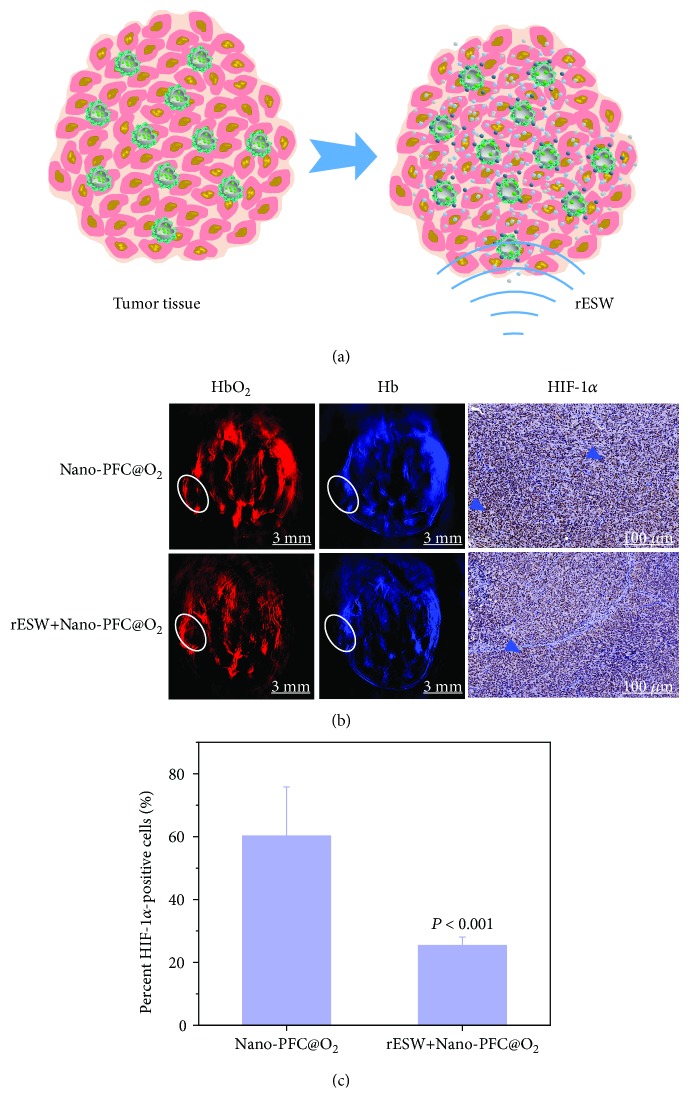
Determination of oxygen release from nanovehicles with the aid of rESW. (a) Schematic illustration of rESW-respective oxygen release from oxygen-saturated Nano-PFC in 4T1 tumor-bearing mice. (b) PA imaging of 4T1 tumors for determining tumor oxygenation status by measuring the ratios of oxygenated hemoglobin (*λ* = 850 nm) and deoxygenated hemoglobin (*λ* = 750 nm) before and after rESW treatments (20 min). Scale bars are 3 mm. The expression levels of HIF-1*α* in the tumors of mice from various groups were analyzed by the immunohistochemical method. Dark blue arrowheads point at the HIF-1*α*-positive cells. Scale bars are 100 *μ*m. (c) Quantification of HIF-1*α*-positive cells in immunohistochemical images by ImageJ software.

Inspired by the above great performance *in vitro*, we further researched the oxygen release behavior *in vivo* based on a 4T1 tumor-bearing mouse model. It is worth mentioning that nanosized materials can achieve highly efficient accumulation in tumor tissue depending on the enhanced permeability and retention (EPR) effect [40]. Additionally, almost all of the solid tumors have a hypoxic region due to the deficient blood supply along with the tumor progression [41, 42]. Likewise, the disturbance of the vascular system is one of the direct reasons for DFUs because it can cause sustained oxygen deficiency and finally chronic hypoxia in the wound area [43]. However, we had established the wound-healing models of DFUs in the rats' forepaws, which were too large to be placed in the detector of the photoacoustic (PA) imaging system. And the scientific rationale is very sound, for the microenvironment, the microstructure, and the pathology are very similar between tumors and diabetic foot ulcers, such as hypoxia, angiogenesis, and inflammation [44–46]. Here, a 4T1 tumor-bearing mouse model was established by subcutaneously injecting 4T1 tumor cells, which belonged to the superficial tumor. And we have proved that rESW could effectively improve local blood flow, which could make more oxygen-loaded Nano-PFC deliveries to the wound location and greater oxygen release under rESW treatment. In support of this finding, our latest study uncovered that rESW could promote vessel vasodilation, tumor blood supply, and nanoparticle extravasation into tumor microenvironment [47]. Therefore, we selected the 4T1 tumor-bearing mouse model as an alternative method to evaluate the *in vivo* behavior of oxygen release and improvement of the hypoxia level as a result of rESW-responsive Nano-PFC. In this process, Nano-PFC can be used as a nanosized carrier system to deliver oxygen into tumor tissue and improve tumor oxygenation under the assistance of the locally applied rESW (Figure 3(a)). The nanodroplets can reversibly release oxygen within the tumor tissue, triggered by the rESW, when Nano-PFC is injected into blood circulation. Thus, the 4T1 tumor-bearing mice breathing pure oxygen were injected with 200 *μ*L Nano-PFC (containing 30 *μ*L of PFC) through the tail vein. Then, the tumor oxygenation status of each mouse was detected by PA imaging after the tumor region was treated with the rESW (1 bar, 4 Hz) for 20 min. PA imaging is noninvasive and is suitable for evaluating the blood oxygenation status by capturing the absorbance of hemoglobin at 750 nm (deoxygenated status) and 850 nm (oxygenated state) [23]. As shown in Figure 3(b), the oxygenation levels over the whole tumor region rapidly increased in the group with hyperoxic breathing plus tumor-localized rESW treatment (rESW+Nano-PFC@O_2_) compared with the group with only hyperoxic breathing (Nano-PFC@O_2_). We also evaluated the expression level of hypoxia-inducible factor 1-alpha (HIF-1*α*), a marker of tumor hypoxia status, by analyzing the immunohistochemistry after 15 days of treatment. In Figures 3(b) and 3(c), the expression levels of HIF-1*α* dropped 58.3% in the rESW+Nano-PFC@O_2_ group in comparison with the Nano-PFC@O_2_ group (*P* < 0.001). These results uncovered the fact that the rESW could effectively stimulate oxygen release from oxygen-loaded Nano-PFC and enhance tumor oxygenation status *in vivo*, which means that the oxygen-loaded Nano-PFC plus rESW could improve the hypoxic microenvironment around DFUs and accelerate the wound healing.

### 3.3. Blood Microcirculation by Laser Doppler Imaging

**Figure 4:**
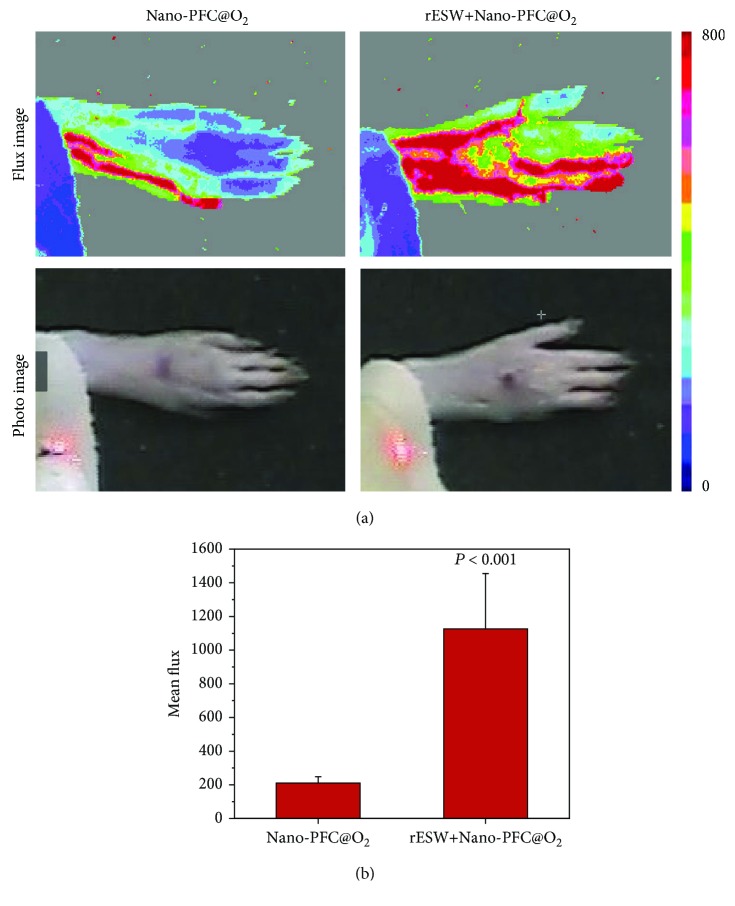
Blood circulation determination of the animal model upon treatment. The change images (a) and quantitative analysis (b) of blood flow in the rat's forepaw detected by laser Doppler imaging measurement for Nano-PFC@O_2_ and rESW+Nano-PFC@O_2_ groups.

Furthermore, it has been proven that the rESW could induce tissue regeneration and improve blood microcirculation [28, 47–49]. Thus, laser Doppler imaging was used to measure the change in blood flow after different treatments. The data revealed that the blood microcirculation of the rat's forepaw was improved about 5.4 times after rESW treatment compared to the Nano-PFC@O_2_ group without the rESW (Figure 4, *P* < 0.001). Nearly every step in the wound healing process requires oxygen for inducing angiogenesis by increasing vascular endothelial growth factor (VEGF) expression [9, 50]. Thus, rESW-responsive improvement of blood microcirculation and oxygen reversible release from Nano-PFC provided the potential for accelerating foot wound healing in STZ-induced DFU models.

### 3.4. Nano-PFC plus rESW Treatment Accelerated Diabetic Foot Wound Healing

**Figure 5:**
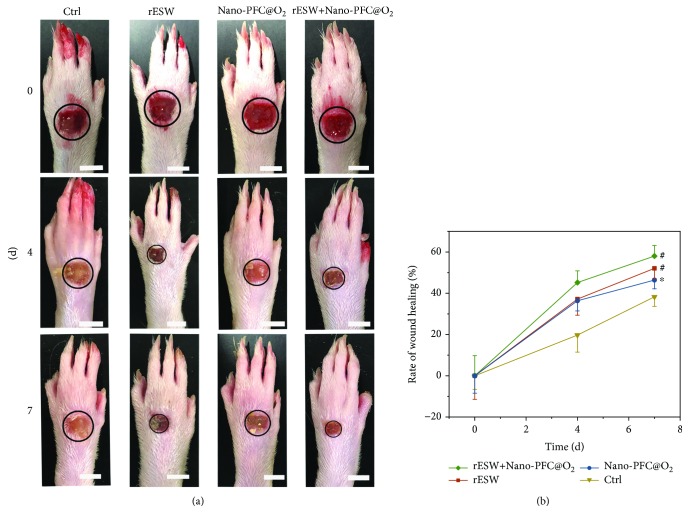
Healing efficacy assessment. Images (a) and size area (b) of the foot wounds in Ctrl, rESW, Nano-PFC@O_2_, and rESW+Nano-PFC@O_2_ groups during the whole treatment. Scale bars are 50 mm.

Nano-PFC@O_2_ and rESW are the key factors in our strategy for the treatment of DFUs. Thus, we further evaluated whether Nano-PFC plus rESW treatment could bring any advantages for the wound healing of DFUs. First, the rat models of STZ-induced DFU were established, and then, a wound was created in each rat's forepaw. As shown in Figure 5, the intervening treatments including rESW, Nano-PFC@O_2_, and rESW plus Nano-PFC@O_2_ inordinately accelerated the wound healing of DFUs, compared to diseased rats with DFUs that did not receive any treatment, namely, the untreated control (Ctrl). Moreover, the wound healing effect of rESW and PFC was also consistent with previous studies [2, 43, 51–57]. And the rate of wound healing in the rESW+Nano-PFC@O_2_ group was significantly higher than that in the Nano-PFC@O_2_ group and rESW group (Figure 5; ^#^ *P* < 0.001 and ^∗^ *P* < 0.05). The improvement of wound healing might be attributed to the rESW and the oxygen supply from Nano-PFC. First, rESW treatment could increase blood microcirculation in the wound healing process. Meanwhile, the oxygen supply from Nano-PFC could neutralize the hypoxic microenvironment due to the vascular disruption and the oxygen consumption by inflammatory and stromal cells within the wound. And rESW treatment might reduce oxidative stress, compared to HBOT, which overcame the drawbacks of HBOT [35, 36].

At present, although many adjunctive therapies have been applied for the healing of DFUs, just as previously discussed [5–7, 12], the clinic treatment of DFUs remained as an enormous challenge due to the multifactors of that. Fortunately, recent studies found that ESW therapy was an effective treatment approach for DFUs, being more effective than HBOT in clinical observations [2, 58, 59]. Moreover, focused ESW was used as a major therapeutic modality in most studies. Thus, herein, we garnered a new sight into the treatment of DFUs. On the other hand, PFC-based bioagents have been shown to be effective in the treatment of chronic wound [43, 51, 60]. Importantly, PFC has been clinically approved by FDA [23, 61]. Based on the above desirable features, we therefore developed the rESW for DFU treatment in combining oxygen-saturated Nano-PFC, which brought a completely new treatment strategy for DFUs. To this end, this strategy provided a great potential for further clinical trials. However, the potential safety issues of PFC-based micro/nanomaterials should not be neglected. Thus, we will continue this study forward.

## 4. Conclusions

In conclusion, the Nano-PFC developed herein exhibited uniform size and achieved the abilities of high-efficiency oxygen loading and rESW-responsive oxygen reversible release *in vitro* and *in vivo*. More importantly, the synergistic effect of the rESW and Nano-PFC accelerated foot wound healing in STZ-induced type 1 diabetes mellitus rats by improving blood microcirculation and providing a targeted supply of oxygen to the wound. This study proved the benefits of combining rESW and Nano-PFC to treat DFUs, which is a novel strategy for the treatment of diabetic foot ulcers.

## Data Availability

The data used to support the findings of this study are available from the corresponding author upon request.

## Supplementary Materials

Supplementary Materials Supplementary description. Table S1: references related to the wound healing studies of diabetic foot ulcers.
