# Supplementary material for: Improved Healing of Diabetic Foot Ulcer upon Oxygenation Therapeutics through Oxygen-Loading Nanoperfluorocarbon Triggered by Radial Extracorporeal Shock Wave
Source: Oxid Med Cell Longev. 2019 Aug 14;2019:5738368. doi: 10.1155/2019/5738368 (PMC6710755; doi:10.1155/2019/5738368)
Supplement: Supplementary Materials — Supplementary description. Table S1: references related to the wound healing studies of diabetic foot ulcers. [file 5738368.f1.pdf]

Supplementary Table S1

Table S1. References related to the wound healing studies of diabetic foot ulcers.[1-8]

Reference	Species	STZ dose (mg/kg)	Period (d)	Blood glucose level (mg/dL)
Theranostics. 2019, 9, 477-490	Male C57BL/6 mice (18-22 g)	60	5	250
J Diabetes Res. 2019, 2019, Article ID 1897174	Male Sprague-Dawley rats	65	14	~540
Wound Repair Regen. 2019, 27, 69-79	Male Wistar rats (4 months old)	50	7	300
Int Wound J. 2019, 16, 144-152	CD hairless rats (9-to10-week-old)	65	-	350
Sci Rep. 2017, 7, 11631	Female albino Wistar rats (5-day-old)	70	63	300
J Diabetes Res. 2016, 2016, Article ID 5782904	Male Sprague-Dawley rats (300–325 g)	70	28	~570
Drug Des Dev Ther. 2016, 2016, 1715-1730	Male Wistar rats (150-250 g)	65	3	~180
Comp Med. 2012, 62, 37-48	Male Wistar rats (250-350 g)	65	8	250

References

[1] R.-F. Chen, C.-H. Chang, C.-T. Wang et al., "Modulation of vascular endothelial growth factor and mitogen-activated protein kinase-related pathway involved in extracorporeal shockwave therapy accelerate diabetic wound healing" Wound Repair and Regeneration, vol. 27, pp. 69-79, 2019.

[2] J.J. Mendes, C.I. Leandro, D.P. Bonaparte and A.L. Pinto, "A rat model of diabetic wound infection for the evaluation of topical antimicrobial therapies" Comparative Medicine, vol. 62, pp. 37-48, 2012.
[3] A.A. Muhammad, P. Arulselvan, P.S. Cheah, F. Abas and S. Fakurazi, "Evaluation of wound healing properties of bioactive aqueous fraction from Moringa oleifera Lam on experimentally induced diabetic animal model" Drug Design, Development and Therapy, vol. 10, pp. 1715-1730, 2016.
[4] J. Ning, H. Zhao, B. Chen et al., "Argon Mitigates Impaired Wound Healing Process and Enhances Wound Healing In Vitro and In Vivo" Theranostics, vol. 9, pp. 477-490, 2019.
[5] S. Qiu, Y. Jia, Y. Sun et al., "Von Hippel-Lindau (VHL) Protein Antagonist VH298 Improves Wound Healing in Streptozotocin-Induced Hyperglycaemic Rats by Activating Hypoxia-Inducible Factor- (HIF-) 1 Signalling" Journal of diabetes research, vol. 2019, pp. Article ID 1897174, 2019.
[6] M. Saidian, J.R.T. Lakey, A. Ponticorvo et al., "Characterisation of impaired wound healing in a preclinical model of induced diabetes using wide-field imaging and conventional immunohistochemistry assays" International wound journal, vol. 16, pp. 144-152, 2019.
[7] C.O.-L. Yu, K.-S. Leung, J.L. Jiang et al., "Low-Magnitude High-Frequency Vibration Accelerated the Foot Wound Healing of n5-streptozotocin-induced Diabetic Rats by Enhancing Glucose Transporter 4 and Blood Microcirculation" Scientific Reports, vol. 7, pp. 11631-11631, 2017.
[8] Y. Zhang, S.A. McClain, H.-M. Lee et al., "A Novel Chemically Modified Curcumin “Normalizes” Wound-Healing in Rats with Experimentally Induced Type I Diabetes: Initial Studies" Journal of Diabetes Research, vol. 2016, pp. Article ID 5782904, 2016.
